# The role of surgery in stage I to III small cell lung cancer: A systematic review and meta-analysis

**DOI:** 10.1371/journal.pone.0210001

**Published:** 2018-12-31

**Authors:** Tingting Liu, Zihao Chen, Jun Dang, Guang Li

**Affiliations:** Department of Radiation Oncology, The First Hospital of China Medical University, Shenyang, Liaoning, China; Rutgers University, Robert Wood Johnson Medical School, UNITED STATES

## Abstract

**Background:**

The role of surgery in treating small cell lung cancer (SCLC) remains controversial. This meta-analysis aims to determine whether surgical-based treatment improves survival in comparison to radiotherapy, chemotherapy, and chemoradiotherapy for stage I to III SCLC.

**Methods:**

PubMed, PubMed Central, EMBASE, Web of Science, and Cochrane Library were searched for relevant articles. The main outcome were overall survival (OS), reported as hazard ratios (HRs), and 95% confidence intervals (CIs).

**Results:**

Two randomized control trials (RCTs) and 13 retrospective studies that included a total of 41,483 patients were eligible. Surgical resection significantly improved OS when compared to non-surgical treatment in retrospective studies (HR = 0.56, 95% CI: 0.49–0.64, P < 0.001), but not in the 2 “older” RCTs (HR = 0.77, 95% CI: 0.32–1.84, P = 0.55). In the subgroup analysis for retrospective studies, surgical resection was associated with superior OS in stage I (HR = 0.56, 95% CI: 0.49–0.64, P < 0.001), stage II (HR = 0.75, 95% CI: 0.57–0.99, P = 0.04), and stage III diseases (HR = 0.70, 95% CI: 0.56–0.88, P = 0.002). Sublobar resection resulted in worse OS than a lobectomy (HR = 0.64, 95% CI: 0.56–0.74, P < 0.001) for patients undergoing surgical resection.

**Conclusions:**

Surgery-based multi-modality treatment appears to be associated with a favorable survival advantage in stage I and selected stage II to III SCLC. Lobectomy is likely to provide superior OS when compared to sublobar resection. Further prospective RCTs are needed to confirm these findings.

## Introduction

Small cell lung cancer (SCLC) represents approximately 13–15% of all lung cancers [1, 2], and is characterized by rapid growth, early development of metastases, and poor prognosis [3]. The standard of care in most patients with stage II to III disease is the combination of platinum-based chemotherapy and thoracic radiotherapy (RT), followed by prophylactic cranial irradiation (PCI) [4, 5]. However, despite initial response to therapy, local recurrences are reported as high as 50% [6, 7], and the overall prognosis is poor. Median survival is 15–20 months with a 2-year survival at 5% [8]. Historically, surgery has not been recommended in treating SCLC mainly due to the findings of 2 randomized controlled trials (RCTs) that reported no survival benefit from surgery when compared to non-operative management [9, 10]. However, these RCTs were performed more than 2 decades ago, which from today’s perspective, did not fulfil modern quality requirements. Recent data from numerous retrospective studies, including some large retrospective cohort studies, have demonstrated a potential survival benefit from surgery in patients with limited disease [11–21]. In light of these findings, we carried out a systematic review and meta-analysis of the currently available evidences to further determine whether surgery-based multi-modality treatment improves survival when compared to RT, chemotherapy, or a combination of both in patients with stage I to III SCLC. Survival differences between lobectomy and sublobar resection were also investigated.

## Methods

This meta-analysis was conducted in accordance with the Preferred Reporting Items for Systematic Reviews and Meta-Analyses (PRISMA) criteria [22] (S1 Table).

### Literature search strategy

PubMed, PubMed Central, EMBASE, Web of Science, and Cochrane Library were searched for available articles published before October 1, 2018, using the following strategy: (((small cell lung cancer [Title/Abstract]) OR (small cell lung carcinoma [Title/Abstract])) NOT ((non-small cell lung cancer [Title/Abstract]) OR (non-small cell lung carcinoma [Title/Abstract]))) AND ((surgery [Title/Abstract]) OR (surgical [Title/Abstract])). Further details of the search strategy are shown in S2 Table. All published papers with available full texts were retrieved. Reference lists of the retrieved articles were manually scanned for relevant additional studies missed by the electronic search.

### Inclusion and exclusion criteria

Studies were included if they met the following criteria: (1) types of studies: RCT, or prospective or retrospective cohort study; (2) types of participants: participants with a cytological or histopathological diagnosis of SCLC and stage I to III disease; (3) types of interventions: compared surgical resection alone or non-surgical treatment in combination with any other therapy including RT, chemotherapy, or a combination of both; (4) outcome: reported overall survival (OS). If multiple articles covered the same study population, the study with the most recent or complete survival data was used. Articles were excluded if any of the following criteria were found: (1) letters, editorials, case reports, and reviews; (2) survival data could not be extracted from the literature.

### Data extraction

The data was extracted by 2 investigators independently, and consensus was reached in case of any discrepancy for all data. The following data was extracted from each study: first author, year of publication, duration of the study, country of origin, number of patients (with and without surgery), study design, treatment, and hazard ratios (HRs) for OS, as well as their 95% confidence intervals (CIs). In case studies did not report sufficient data, the authors of those studies were contacted for further information via e-mail if possible.

### Quality assessment

The Newcastle-Ottawa Quality Assessment Scale (NOS) was used to assess the quality of the retrospective studies [23]. The NOS comprises of 3 items: patient selection, comparability of the study groups, and assessment of outcomes. The quality of each cohort study was scored on a scale of 0–9 by 2 independent researchers. Studies with six stars or greater were considered to be sufficiently high-quality studies.

The methodological quality of RCTs was assessed using the Cochrane risk of bias tool [24], which consists of the following 5 domains: sequence generation, allocation concealment, blinding, incomplete data, and selective reporting. They were finally rated as “low risk of bias” (all key domains indicated as low risk), “high risk of bias” (one or more key domains indicated as high risk), and “unclear risk of bias”.

### Statistical analysis

Statistical analysis was performed using the Review Manager 5.3 (Cochrane Collaboration, Oxford, UK) and STATA MP 14.0 (Stata Corporation, College Station, TX, USA) softwares. Since median survival or survival rates at a specific point in time were not expected to be reliable surrogate measures for pooled survival analysis, HRs and their 95% CIs were used as summary statistics for OS in the present meta-analysis. Crude HRs with 95% CIs were either extracted directly from the original reports or calculated using Kaplan-Meier curves based on the methods of Parmer et al. [25] and Tierney et al. [26]. A statistical test for heterogeneity was performed via the Chi-square (χ^2^) and I-square (I^2^) tests with significance set at P < 0.10 and/or I^2^ > 50%, respectively. If significant heterogeneity existed, a random-effects analysis model was used; otherwise, a fixed-effects model was used. Cumulative meta-analysis was performed to assess the evolution of OS in time. We also conducted subgroup and meta-regression analysis to search for the source of heterogeneity. The stability of the pooled results was evaluated via a sensitivity analysis in which the data of an individual study was removed each time. A funnel plot, Begg’s test [27], and Egger’s linear regression test [28] were performed to investigate any potential publication bias. If evidence of publication bias was observed, the trim-and-fill method was applied to correct the bias. P < 0.05 was considered to be statistically significant.

## Results

### Literature search results and characteristics of included studies

**Fig 1:**
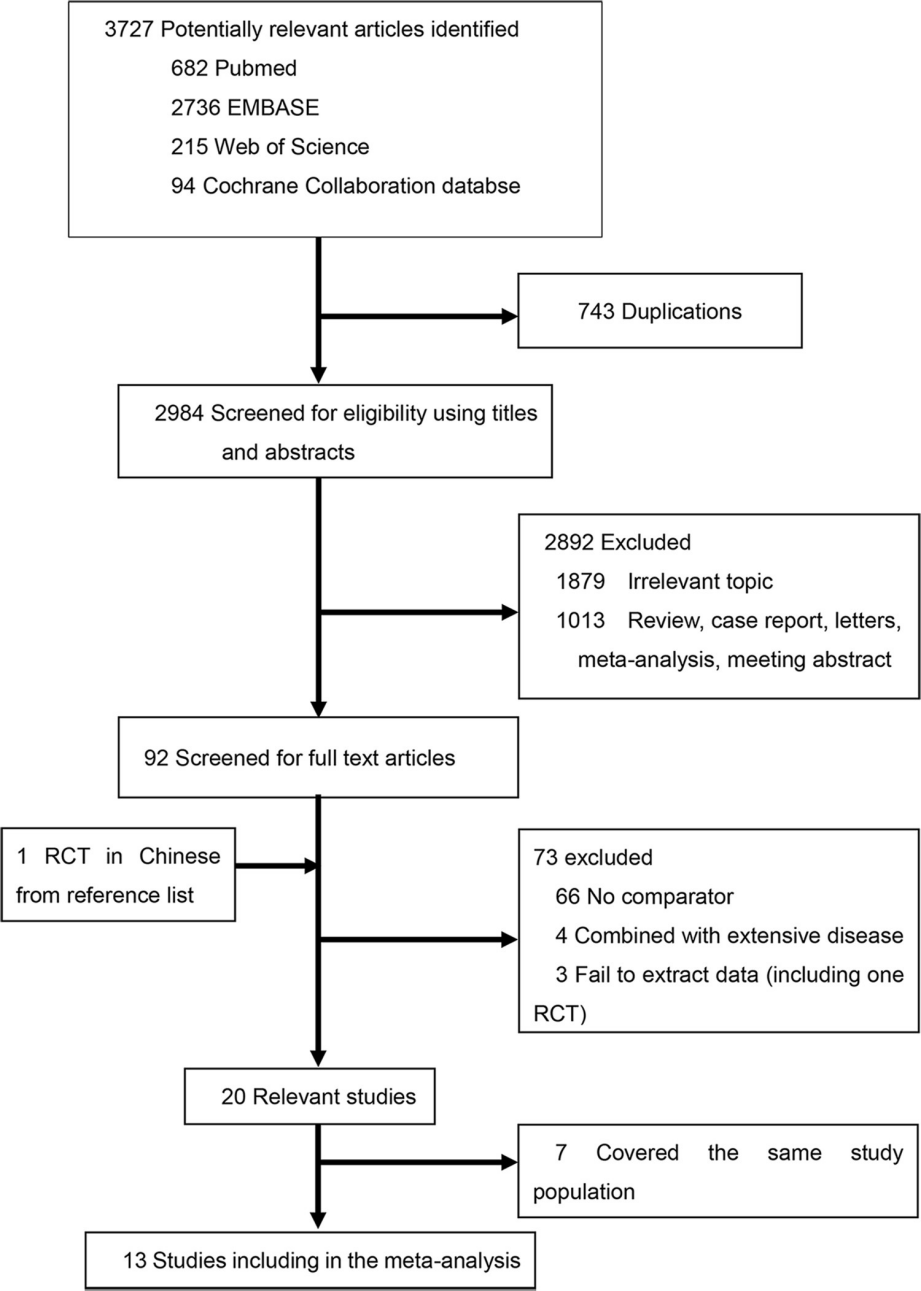
Literature search and selection.

The literature search and study selection procedures are shown in Fig 1. The initial search from the electronic database retrieved 3,727 articles. After removing the duplicates, 2,984 citations were identified. Of these, 2,892 were excluded via an abstract review and the remaining 92 articles were screened via a full-text review for further eligibility. Twenty articles were identified as potentially relevant. Since 5 Surveillance, Epidemiology, and End Results (SEER)-based articles and 6 National Cancer Data Base (NCDB)-based articles covered the same study population, 7 articles were excluded and 4 with the most recent and complete survival data were retained. Finally, 15 articles (2 RCTs and 13 retrospective studies) assessing 41,483 patients (4,970 patients receiving surgery-based treatment and 36,513 patients receiving non-surgical treatment) were included in the meta-analysis. Characteristics of the eligible studies are summarized in Table 1.

**Table 1:** Baseline characteristics of included studies.

First author/ Year	Country of origin	Study language	Time range	Patients (n) (Surgery/NST)	Study design	NOS score
Lad/1994 [10]	Europe	English	NR	70/76	RCT	-
Liao/1995 [29]	China	Chinese	1990–1991	20/20	RCT	-
Wakeamet/2017 [11]	United States	English	2004–2013	2089/2089	RS	7
Ahmed/2017 [12]	United States	English	2007–2013	543/815	RS	7
Schreiber/2010 [13]	United States	English	1988–2002	863/13316	RS	7
Combs/2015 [14]	United States	English	1998–2011	663/16089	RS	7
Zhu/2013 [15]	China	English	1996–2006	96/49	RS	7
Badzio/2004 [16]	Poland	English	1984–1996	67/67	RS	6
Zhang/2014 [17]	China	English	1995–2013	50/103	RS	7
Hou/2017 [18]	China	English	2005–2010	102/106	RS	6
Takenaka/2015 [19]	Japan	English	1974–2011	88/50	RS	7
Yin/2018 [20]	China	English	2010–2015	70/70	RS	7
Chen/2018 [21]	United States	English	2004–1014	176/3586	RS	7
Ichinose/1992 [30]	Japan	English	1974–1989	37/32	RS	7
Hara/1991 [31]	Japan	English	1972–1989	36/45	RS	6

Abbreviations: NST: non-surgical treatment; RCT: randomized controlled trial; RS: retrospective cohort study; NOS: Newcastle-Ottawa Quality Assessment Scale score.

### Assessment of included studies

Both researchers showed good consistency in assessing the quality of the 15 included studies (Table 1). All the retrospective studies demonstrated a score ≥ 6 (S3 Table). The qualities of the included RCTs were generally low. One RCT [10] was considered to be “high risk” and the other [29] was classified as “unclear” with respect to risk of bias (S4 Table).

### Comparison of OS between the surgery and non-surgical treatment (NST) groups

**Fig 2:**
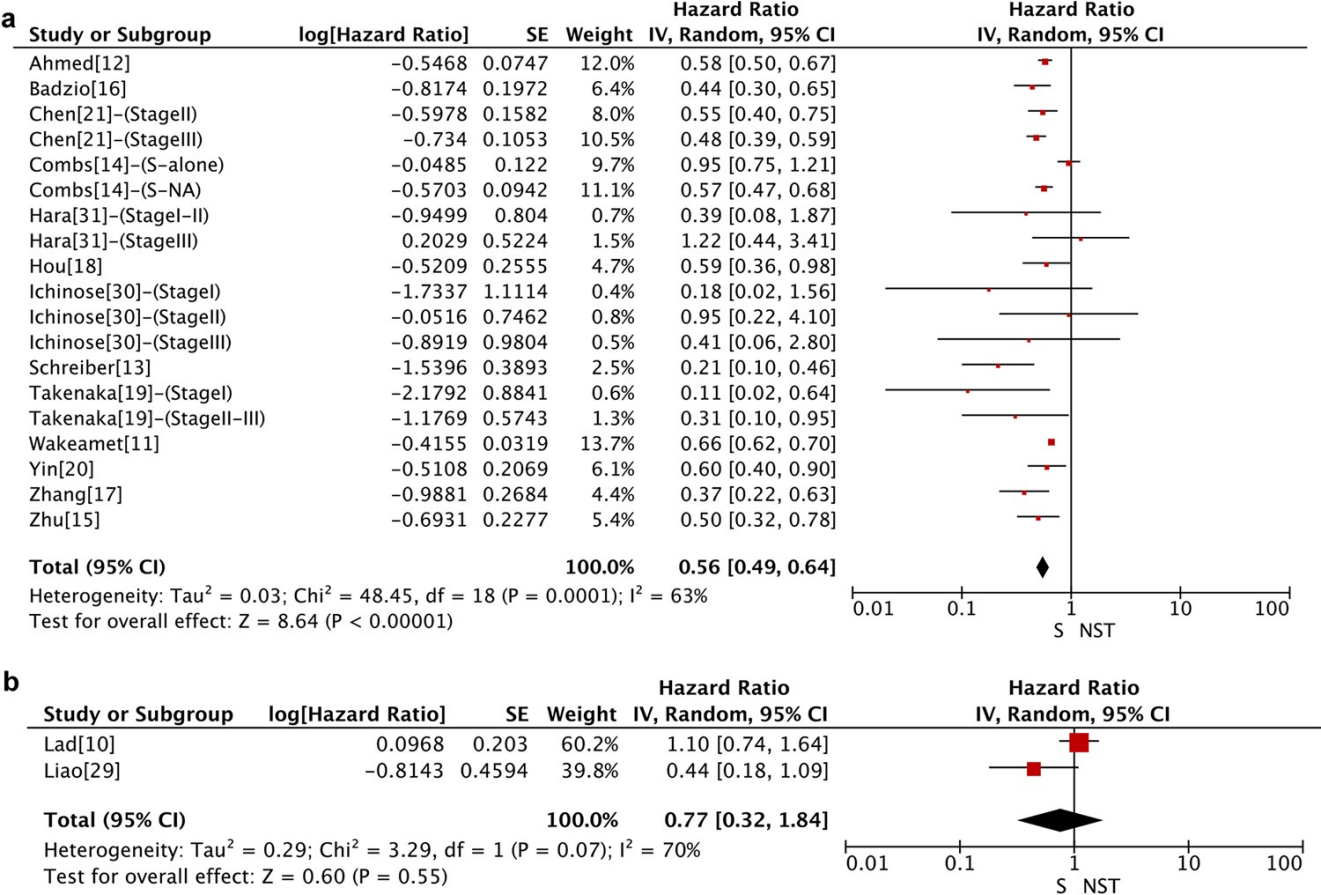
Forest plots of HR for OS in (a) retrospective studies and (b) RCTs. RCTs: randomized controlled trials; NST: non-surgical treatment; OS: overall survival; CI: confidence interval; SE: standard error; IV: inverse variance method.

**Fig 3:**
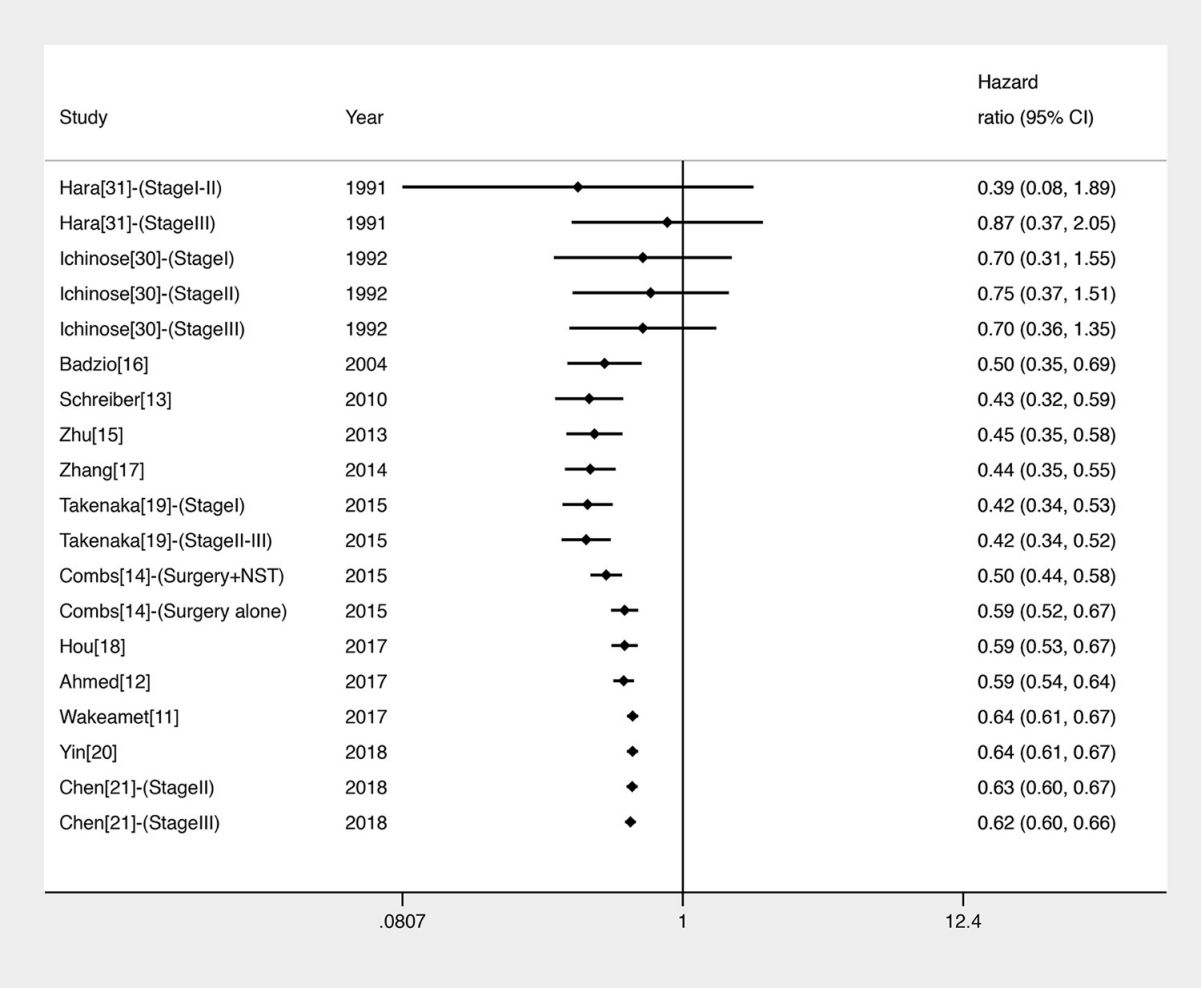
Cumulative meta-analysis for the comparison of OS between surgical and non-surgical treatments in retrospective studies. NST: non-surgical treatment.

We divided the 15 articles into 21 studies because 5 articles were straight stratified according to surgical treatment type or clinical stage [14, 19, 21, 30–31]. HRs were extracted directly for 7 out of 15 studies [11, 14–17, 20–21], and calculated using the Kaplan-Meier curve for the remaining. Significant statistical difference was observed between the surgery and NST groups in a pooled analysis of OS for 41,297 patients from retrospective studies (HR = 0.56, 95% CI: 0.49–0.64, P < 0.001) (Fig 2A), but not for 186 patients from RCTs (HR = 0.77, 95% CI: 0.32–1.84, P = 0.55) (Fig 2B). Heterogeneity was evident across the studies (I^2^ = 63%, P < 0.001 for retrospective studies; I^2^ = 70%, P = 0.07 for RCTs). For the included studies that had a time span over 20 years, a cumulative meta-analysis of the data related to OS was carried out in retrospective studies where these studies were added in order of the publication date (Fig 3). Publications since 2004 [11–21] were observed to report statistically significant and unchanged OS benefit in the surgery group.

Results of the subgroup analysis for retrospective studies are listed in Table 2. Except for the studies published before 2004 (HR = 0.70, 95% CI: 0.36–1.35, P = 0.29; P_heterogeneity_ = 0.45) and surgery alone (HR = 0.87, 95% CI: 0.71–1.06, P = 0.16; P_heterogeneity_ = 0.01) subgroups, surgery was associated with significantly improved OS for sample sizes ≥ 100 (HR = 0.56, 95% CI: 0.49–0.64, P < 0.001; P_heterogeneity_ < 0.001), sample sizes < 100 (HR = 0.49, 95% CI: 0.28–0.83, P = 0.009; P_heterogeneity_ = 0.22), studies published after 2004 (HR = 0.55, 95% CI: 0.48–0.63, P < 0.001; P_heterogeneity_ < 0.001), surgery + NST (HR = 0.60, 95% CI: 0.53–0.67, P < 0.001; P_heterogeneity_ = 0.06), stage I (HR = 0.56, 95% CI: 0.49–0.64, P < 0.001; P_heterogeneity_ = 0.05), stage II (HR = 0.75, 95% CI: 0.57–0.99, P = 0.04; P_heterogeneity_ = 0.006), and stage III (HR = 0.70, 95% CI: 0.56–0.88, P = 0.002; P_heterogeneity_ < 0.001).

**Table 2:** Subgroup and meta-regression analysis of the effect on OS from surgical treatment.

Subgroup	Included studies No. [References]	No. of Patients (Surgery/NST)	HR [95% CI]	Heterogeneity		Meta-regression
				I^2^ (%)	P-Value	P-Value
Sample size						0.61
≥ 100	12 [11–18,20–21]	4719/36290	0.56 [0.49–0.64]	72	< 0.001	
< 100	7 [19,30–31]	161/127	0.49 [0.28–0.83]	27	0.22	
Publication date						0.58
Before 2004	5 [30–31]	73/77	0.70 [0.36–1.35]	0	0.45	
After 2004	14 [11–21]	4807/36340	0.55 [0.48–0.63]	71	< 0.001	
Surgical treatment type						0.01
Surgery + NST	15 [11–12,14–18,20,30–31]	3299/19403	0.60 [0.53–0.67]	39	0.06	
Surgery alone	5 [11–12,14]	857/18950	0.87 [0.71–1.06]	70	0.01	
Clinical stage						0.16
Stage I	6 [11–12,14,16,19,30]	2429/4746	0.56 [0.49–0.64]	54	0.05	
Stage II	8 [11,14–16,19–20–21,30]	613/3550	0.75 [0.57–0.99]	64	0.006	
Stage III	10 [11,13–14,16–17,19–21,30–31]	917/22542	0.70 [0.56–0.88]	74	< 0.001	

Abbreviations: NST: non-surgical treatment.

Meta-regression analysis was performed to investigate the potential source of heterogeneity among retrospective studies (Table 2).The results demonstrated that the surgical treatment type, surgery + NST/surgery alone (P = 0.01), was the evident contributor to heterogeneity.

**Fig 4:**
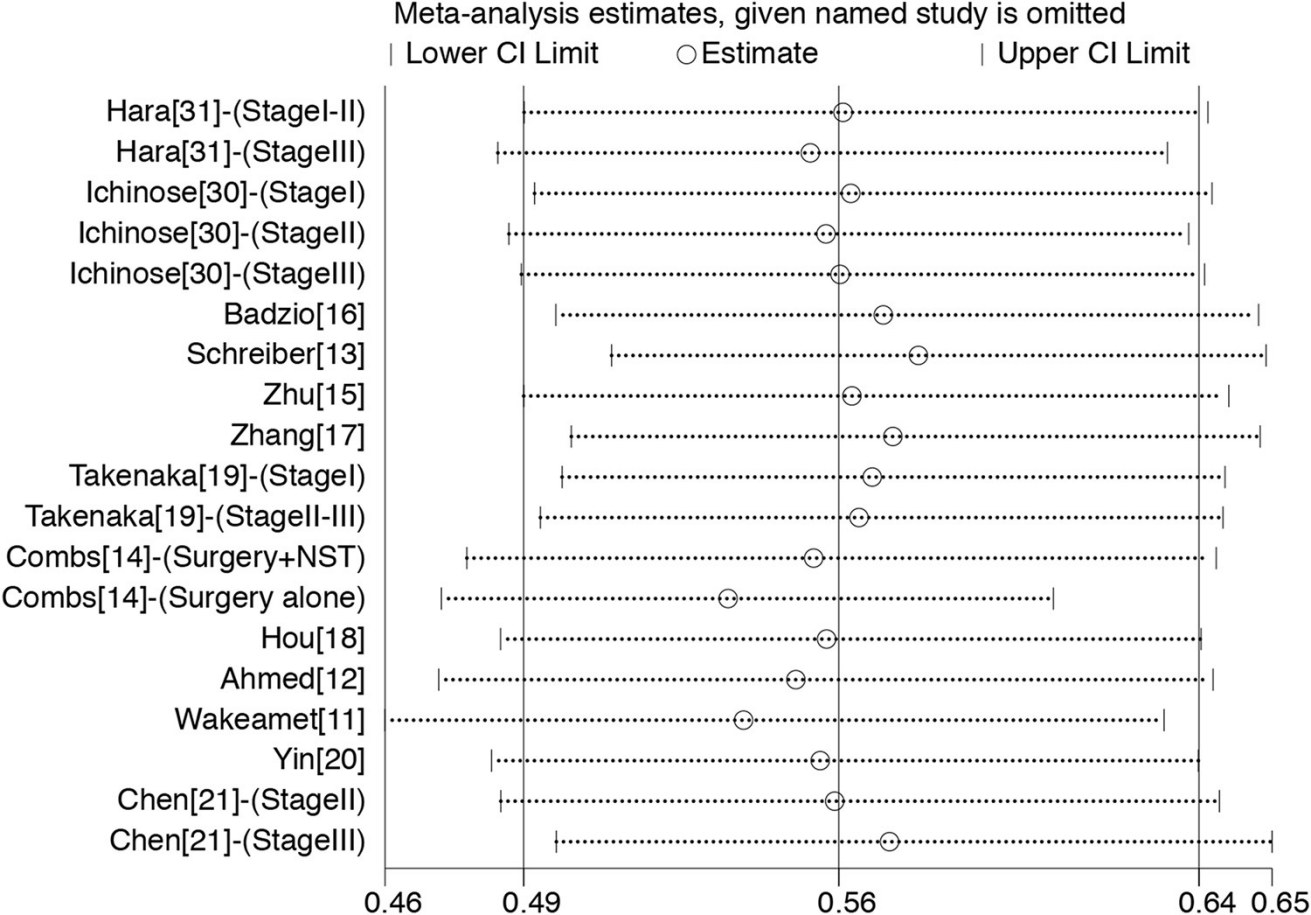
Sensitivity analysis for the comparison of OS between surgical and non-surgical treatments in retrospective studies.

Sensitivity analyses were also carried out to assess whether individual studies influenced the results (Fig 4). When individual studies were removed one at a time from the analyses, the corresponding pooled HRs were not markedly altered by any single study, indicating the stability of the presented results.

### Comparison of OS between the lobectomy and sublobar resection groups

**Fig 5:**
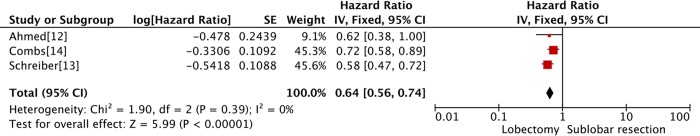
Forest plots of HR for OS with respect to lobectomy versus sublobar resection. OS: overall survival; CI: confidence interval; SE: standard error; IV: inverse variance method.

Sublobar resection resulted in worse OS than lobectomy (3 studies with 2,691 patients; HR = 0.64, 95% CI: 0.56–0.74, P < 0.001), and heterogeneity was not found to be significant (I^2^ = 0%, P = 0.39) (Fig 5).

### Assessment of publication bias

**Fig 6:**
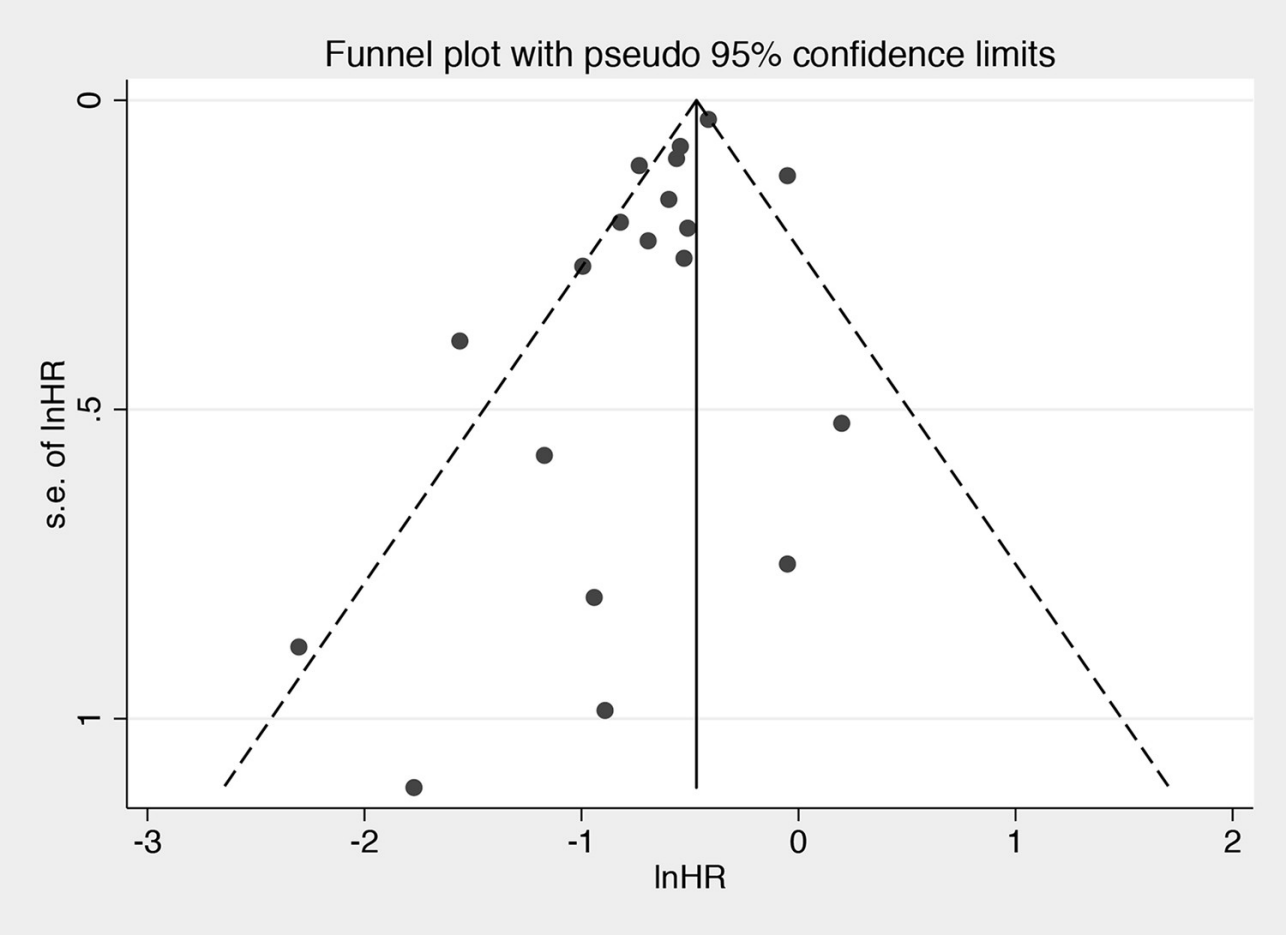
Funnel plot of all studies with a pseudo 95% confidence interval (CI).

Publication bias in terms of OS was assessed in retrospective studies. The funnel plot is shown in Fig 6. Although the Begg’s test results indicated no publication bias (P = 0.62), Egger’s test suggested a significant probability of publications bias (P = 0.03). However, the trim-and-fill method demonstrated that no missing studies were detected, indicating that our results were reliable.

## Discussion

To our knowledge this is the only meta-analysis to evaluate the use of surgery in the management of stage I to III SCLC. This meta-analysis enrolled 2 “older” RCTs and 13 retrospective studies with 41,483 patients. Pooled analysis of the OS for 41,297 patients from retrospective studies showed that the OS achieved via surgery-based strategies was higher than non-surgical treatment. However, no significant difference in OS was observed between the surgery and NST groups for 186 patients from 2 RCTs. There was significant heterogeneity among retrospective studies. Based on subgroup and meta-regression analysis, surgical treatment type (surgery + NST/surgery alone) was identified as the evident contributor to heterogeneity. Moreover, results of sensitivity analysis showed that the corresponding pooled HRs were not markedly altered by any single study when individual studies were removed one at a time from the analyses, indicating the stability of the presented results.

A recent review by Barnes et al., which evaluated surgery versus non-operative management for SCLC [32], examined 3 RCTs [9, 10, 29] and concluded that the current evidences did not support a role for surgical resection in the management of limited-stage SCLC. An RCT conducted by Fox et al. in 1973 [9] showed poor mean survival for the surgical group when compared to the RT group (6.5 months vs. 10 months, P = 0.04). The other RCT performed by Lad et al. in 1994 [10] showed no significant difference in survival between the groups. The last one performed by Liao et al. in 1995 [29] found a higher survival rate in the surgical group when compared to the RT group; however, it was not statistically significant. These results showed that there was no role for surgery in the multimodality treatment of SCLC. However, these RCTs were of low quality and had some limitations. For example, chemotherapy was not included as a part of the standard treatment protocol and only 34 out of 71 participants underwent surgical resection in the surgical arm in the Fox trial, whereas the Lad trial only included participants with regional nodal involvement.

There have been many developments in SCLC therapy since these trials, such as etoposide- and cisplatin-based chemotherapy, modern RT techniques, radiation given concurrently with chemotherapy, and better diagnostic and surgical tools. Therefore, it is timely and appropriate to rediscuss the treatment algorithm for SCLC, especially with regard to the potential contribution of surgery in limited-stage SCLC. Data from a series of recent observational studies have supported surgical intervention in the management of limited-stage SCLC [11–21]. Our cumulative meta-analysis found that the OS benefit in the surgical-group was statistically significant and unchanged since the publication of the 2004 studies. The current National Comprehensive Cancer Network (NCCN) guidelines recommend surgical resection for clinical stage I (T1-2, N0) disease, despite the fact that these recommendations are based on limited data [33]. In the present meta-analysis, significantly improved OS was observed in patients receiving surgery-based treatments when compared to patients receiving non-surgical treatments in the stage I SCLC subgroup.

Unlike stage I disease, there is no consensus for surgery in stage II and stage IIIA SCLC. Current NCCN guidelines state that patients with disease exceeding T1-T2, N0 do not benefit from surgery [33]. However, several recently published population-based studies [11, 13, 34] have shown that surgery was significantly associated with improved survival in patients with stage II and stage IIIA SCLC. Unexpectedly, the present meta-analysis of retrospective studies also showed survival benefits from surgery for stage II and IIIA disease. In individual studies, surgical patients with stage II and IIIA who had longer survival were more likely to receive lobectomy and/or receive adjuvant chemoradiation [11, 13–14]. Yin et al. [20] retrospectively assessed the efficacy of surgery in patients with stage II to IIIA SCLC and found a marginal OS benefit from surgery. However, further subgroup analysis of stage IIIA showed significantly improved OS from surgery in patients who received adjuvant chemoradiation and PCI (P = 0.01). These results suggested a possible role of surgery in selected stage II and IIIA SCLC treatment. Notably, many of the studied patients in the non-surgical groups were staged according to thoracic CT, abdominal ultrasonography, and bronchoscopy without modern tools such as positron emission tomography-computed tomography (PET-CT) or mediastinoscopy. Thomson et al. reported that with modern series PET-CT, a median 9% of the patients were upstaged and 4% were downstaged [35]. Thus, drawing conclusions based on clinical stage to determine surgical treatment effects on OS should be discreet. Nevertheless, these recent evidences raise important questions regarding surgical intervention in limited stage SCLC and support undertaking a randomized, prospective clinical trial to answer these questions.

In this meta-analysis, surgical resection saw significant improvement in OS in the surgery + NST subgroup, but the surgery alone subgroup was identified as a source of heterogeneity. Surgery + NST has been reported to provide superior OS in numerous individual studies [11–12, 14–18]. Results from an NCDB-based study showed significant differences in the 5-year OS for treatment groups in all clinical stages, with surgery + chemotherapy having the best OS [14]. Moreover, Wakeam et al. reported that surgical patients not receiving adjuvants did worse than their matched non-surgical counterparts at stages I and IIIA [11]. These results suggested that surgical resection in combination with chemotherapy and/or RT could be crucial in improving the survival of patients with resectable SCLC.

Lobectomies are the standard of care for the resection of stage I NSCLC, providing higher survival and lower risk of local recurrence than sublobar resections. Recently, superior outcomes for lobectomies were frequently reported in stage I SCLC treatment. Data from a retrospective NCDB review showed that the 5-year survival for lobectomies was better than sublobar resections (49% vs. 30% for stage I disease and 40% vs. 21% for all limited stages) [14]. Similarly, results from the SEER database showed that the median survival was 40 months for lobectomy resections and 23 months for sublobar resections in all limited stage cases [13]. It also showed that sublobar resections resulted in a worse 5-year OS rate when compared to lobectomies (34% vs. 50%) in a subset of stage I patients [36]. Consistent with these results, lobectomies showed superior OS when compared to sublobar resections in our meta-analysis. These findings suggest that lobectomies may be more useful as surgery-based multi-modality treatments for SCLC.

Unfortunately, our meta-analysis has some limitations. Firstly, the study included only 2 RCTs and almost all the available data was extracted from retrospective studies. The observational data had inherent limitations, such as clinical factors were potentially imbalanced; and the RT technique, RT doses, or chemotherapy schedules used in the individual studies were different, which inevitably led to heterogeneity. Secondly, most of the studied patients were staged according to thoracic CT, abdominal ultrasonography, and bronchoscopy without modern tools such as PET-CT or mediastinoscopy, which led to less accurate clinical staging. Thirdly, the impact of adjuvant chemotherapy or RT on OS was not evaluated as some studies reported them individually. Fourthly, most of the HRs were not directly reported in the texts (8 out of 15) and hence, had to be calculated using the Kaplan-Meier curve. This may have resulted in bias and error. Finally, PCI may have been a confounding factor for OS in this study. The benefit of PCI on long-term survival is recognized and has been recommended by the NCCN for selected patients [33]. PCI was reported to be used in some included studies. However, we could not assess the effect of PCI on OS between surgical and non-surgical groups for lack of detailed information in those included studies.

## Conclusions

Surgery-based multi-modality treatment seems to be associated with a favorable survival advantage in stage I and selected stage II to III SCLC. Lobectomy is likely to provide superior OS when compared to sublobar resection. Further prospective randomized controlled trials are needed to confirm these findings.

## Supporting information

S1 Table PRISMA checklist. (DOC)

S2 Table Search strategy. (DOC)

S3 Table Quality assessment of 11 cohort studies using NOS. (DOC)

S4 Table Quality assessment of 2 RCT studies using the Cochrane risk of bias tool. (DOC)
