# Supplementary material for: The role of surgery in stage I to III small cell lung cancer: A systematic review and meta-analysis
Source: PLoS One. 2018 Dec 31;13(12):e0210001. doi: 10.1371/journal.pone.0210001 (PMC6312204; doi:10.1371/journal.pone.0210001)
Supplement: S2 Table — (DOC) [file pone.0210001.s002.doc]

**S2 table Search strategy**

**a:** Search strategy in PubMed

| # | Query |
| --- | --- |
| #1 | “Small Cell Lung Carcinoma”[mh] |
| #2 | Small Cell Lung Cancer[tiab] OR Carcinoma, Small Cell Lung[tiab] OR Small Cell Cancer Of The Lung[tiab] OR SCLC[tiab] |
| #3 | "Carcinoma, Non-Small-Cell Lung"[mh] |
| #4 | non small cell lung cancer[ti] OR NSCLC[tiab] |
| #5 | #1 OR #2 |
| #6 | #3 OR #4 |
| #7 | #5 NOT #6 |
| #8 | “Pneumonectomy”[mh] |
| #9 | “resect*”[tiab] OR “lobectom*”[tiab] OR surgical[tiab] OR surgery[tiab] |
| #10 | #8 OR #9 |
| #11 | #7 AND #10 |

**b:** Search strategy in Embase

| # | Query |
| --- | --- |
| #1 | 'small cell lung cancer'/exp |
| #2 | 'small cell lung cancer':ab,ti OR 'carcinoma, small cell lung':ab,ti OR 'small cell cancer of the lung':ab,ti OR 'sclc':ab,ti |
| #3 | 'non small cell lung cancer'/exp |
| #4 | 'non small cell lung cancer':ti OR 'nsclc':ab,ti |
| #5 | #1 OR #2 |
| #6 | #3 OR #4 |
| #7 | #5 NOT #6 |
| #8 | 'lung resection'/exp |
| #9 | 'resect*':ab,ti OR 'lobectom*':ab,ti OR 'surgical':ab,ti OR 'surgery':ab,ti OR 'surg*':ab,ti OR 'pneumonectom*':ab,ti |
| #10 | #8 OR #9 |
| #11 | #7 AND #10 |

**c:** Search strategy in Cochrane Library

| # | Query |
| --- | --- |
| #1 | MeSH descriptor: [Small Cell Lung Carcinoma] explode all trees |
| #2 | (small cell lung cancer):ti,ab,kw OR (carcinoma, small cell lung):ti,ab,kw OR (small cell cancer of the lung):ti,ab,kw OR (sclc):ti,ab,kw |
| #3 | MeSH descriptor: [Carcinoma,Non-Small-Cell-Lung] explode all trees |
| #4 | (non small cell lung cancer):ti,ab,kw OR (nsclc):ti,ab,kw |
| #5 | #1 OR #2 |
| #6 | #3 OR #4 |
| #7 | #5 NOT #6 |
| #8 | MeSH descriptor: [Pneumonectomy] explode all trees |
| #9 | (resect*):ti,ab,kw OR (lobectom*):ti,ab,kw OR (surgical):ti,ab,kw OR (surgery):ti,ab,kw OR (surg*):ti,ab,kw OR (pneumonectom*):ti,ab,kw |
| #10 | #8 OR #9 |
| #11 | #7 AND #10 |

**d:** Search strategy in Web of Science

| # | Query |
| --- | --- |
| #1 | TS=(“Small Cell Lung Carcinoma” OR "small cell lung cancer" OR SCLC OR “carcinoma, small cell lung” OR “small cell cancer of the lung”) |
| #2 | TS=("non small cell lung cancer" OR "Carcinoma,Non-Small-Cell-Lung" OR "NSCLC") |
| #3 | TS=("Pneumonectomy" OR "resect*” OR “lobectom*” OR “surgical” OR “surgery” OR “surg*” OR “pneumonectom*”) |
| #4 | #1 NOT #2 |
| #5 | #4 AND #3 |
