# Supplementary material for: The role of surgery in stage I to III small cell lung cancer: A systematic review and meta-analysis
Source: PLoS One. 2018 Dec 31;13(12):e0210001. doi: 10.1371/journal.pone.0210001 (PMC6312204; doi:10.1371/journal.pone.0210001)
Supplement: S3 Table — (DOC) [file pone.0210001.s003.doc]

**S3 Table Quality assessment of eleven cohort studies using the Newcastle-Ottawa scale**

| First author/ | Selection | | | |  | Comparablity | |  | Outcome | | | Score |
| --- | --- | --- | --- | --- | --- | --- | --- | --- | --- | --- | --- | --- |
| year | Item1 | Item2 | Item3 | Item4 |  | Item5 | Item6 |  | Item7 | Item8 | Item9 |  |
| Wakeamet/2017 [11] | - | * | * | * |  | * | * |  | * | * | - | 7 |
| Ahmed/2017 [12] | - | * | * | * |  | * | * |  | * | * | - | 7 |
| Schreiber/2010 [13] | - | * | * | * |  | * | * |  | * | * | - | 7 |
| Combs/2015 [14] | - | * | * | * |  | * | * |  | * | * | - | 7 |
| Zhu/2013 [15] | - | * | * | * |  | * | * |  | * | * | - | 7 |
| Badzio/2004 [16]] | - | * | * | * |  | * | - |  | * | * | - | 6 |
| Zhang/2014 [17] | - | * | * | * |  | * | * |  | * | * | - | 7 |
| Hou/2017 [18] | - | * | * | * |  | * | - |  | * | * | - | 6 |
| Takenaka/2015 [19] | - | * | * | * |  | * | * |  | * | * | - | 7 |
| Yin/2018[20] | - | * | * | * |  | * | * |  | * | * | - | 7 |
| Chen/2018[21] | - | * | * | * |  | * | * |  | * | * | - | 7 |
| Ichinose/1992 [30] | - | * | * | * |  | * | * |  | * | * | - | 7 |
| Hara/1991 [31] | - | * | * | * |  | * | - |  | * | * | - | 6 |

Abbreviations: - : zero point; * : one point. Item 1: representativeness of the exposed cohort; Item 2: selection of the non exposed cohort; Item 3: ascertainment of exposure; Item 4: demonstration that outcome of interest was not present at start of study; Item 5: comparability of cohorts on the basis of the design (study controls for the most important factor); Item 6: comparability of cohorts on the basis of the design (study controls for other additional factor); Item 7: assessment of outcome; Item 8: follow-up long enough for outcomes to occur; Item 9: adequacy of follow-up of cohorts.
