# Supplementary material for: The role of surgery in stage I to III small cell lung cancer: A systematic review and meta-analysis
Source: PLoS One. 2018 Dec 31;13(12):e0210001. doi: 10.1371/journal.pone.0210001 (PMC6312204; doi:10.1371/journal.pone.0210001)
Supplement: S4 Table — (DOC) [file pone.0210001.s004.doc]

**S4 Table** Quality assessment of two RCT studies using the Cochrane risk of bias tool

| First author/  year | Random sequence  generation | Allocation  concealment | Double blinding  method | Incomplete outcome  data | Selective  reporting | Other  bias |
| --- | --- | --- | --- | --- | --- | --- |
| Lad/1994 [10] | Unclear | Unclear | Unclear | Low | Low | Unclear |
| Liao/1995 [29] | Unclear | Unclear | Unclear | High | Unclear | Unclear |

Abbreviations: Low: low risk of bias; Unclear: low risk of bias; High: high risk of bias.
